# Tight and stable glucose control is associated with better prognosis in patients hospitalized for Covid-19 and pneumonia

**DOI:** 10.1007/s00592-024-02409-8

**Published:** 2024-11-29

**Authors:** Amelia Caretto, Gaetano Di Terlizzi, Erika Pedone, Renato Pennella, Francesco De Cobelli, Moreno Tresoldi, Marina Scavini, Emanuele Bosi, Andrea Laurenzi

**Affiliations:** 1 Diabetes Research Institute, IRCCS San Raffaele Scientific Institute, Milan, Italy; 2 Department of Internal Medicine, Diabetology, Endocrinology and Metabolism, IRCCS San Raffaele Scientific Institute, Milan, Italy; 3 Unit of General Medicine and Advanced Care, IRCCS San Raffaele Scientific Institute, Milan, Italy; 4 Department of Radiology, IRCCS San Raffaele Scientific Institute, Milan, Italy; 5 University Vita-Salute San Raffaele, Via Olgettina 60, 20132 Milan, Italy

**Keywords:** Continuous glucose monitoring, Diabetes, Covid-19, Tight time in range, SARS-Cov-2, Glucose variability

## Abstract

**Aims:**

To investigate possible associations of glucose patterns with outcomes of Corona Virus Disease 19 (COVID-19) using continuous glucose monitoring (CGM) in 43 patients hospitalized for COVID-19 mild-to-moderate pneumonia, regardless of diabetes.

**Methods:**

Prospective observational study conducted during two pandemic waves in 2020–2021. Glucose sensor metrics of 7-day recording were obtained from blinded CGM. Respiratory function was evaluated as arterial partial pressure of oxygen (PaO_2_) to fraction of inspired oxygen (FiO_2_) ratio (PaO_2_:FiO_2_).

**Results:**

PaO_2_:FiO_2_ ratio was positively correlated with time in tight range (TITR) 70–140 (r = 0.49, p < 0.001) and time in range (TIR) 70–180 (r = 0.32, p < 0.05), and negatively correlated with average glucose (r =– 0.31, p < 0.05), coefficient of glucose variation (CV) (r =– 0.47, p < 0.01) and time above range (TAR) > 140 (r =– 0.49, p < 0.001). No relations were observed with HbA1c. Multivariate regression analysis showed that normal respiratory function at time of CGM removal correlated positively with TITR 70–140 mg/dL (p < 0.01), negatively with CV and TAR > 140 mg/dL (both p < 0.05) and not with TIR 70–180 and average glucose.

**Conclusions:**

Lower glucose variability and optimal glucose control, expressed as CV and TITR, are CGM metrics predictive of a better prognosis in COVID-19 patients with pneumonia.

**Supplementary Information:**

The online version contains supplementary material available at 10.1007/s00592-024-02409-8.

## Introduction

Since the early reports of the pandemic, both diabetes mellitus and incidental hyperglycemia have been identified as predictors of worse clinical outcomes in patients hospitalized for Corona Virus Disease 19 (COVID-19) [1–6]. A first explanation is that type 2 diabetes (T2D), by far the most represented form of diabetes in the large COVID-19 series reports, is preferentially associated with other recognized risky conditions such as older age, hypertension, cardiovascular diseases, overweight and obesity, all negatively influencing the overall prognosis of COVID-19 [7]. However, some observations also indicate a possible role of glucose itself, irrespective of that played by accompanying comorbidities. Some studies have also supposed a direct diabetogenic effect of the severe acute respiratory syndrome coronavirus 2 (SARS-Cov-2) infection [8–11]. Of paramount importance is the evidence that the increased severity and mortality of COVID-19 observed in patients with T2D is equally present in type 1 diabetes (T1D) [12–14], a form of diabetes primarily dominated by glucose abnormalities, with no interferences by the pathogenetic determinants of the T2D associated comorbidities. Furthermore, the prognosis of COVID-19 is worse in diabetic patients with poor control in comparison with those with a better glycemic control [3, 13, 14].

Continuous glucose monitoring (CGM) is a technology for clinical use in people with diabetes, providing accurate information on several glucose metrics, ultimately helpful to optimize therapy and achieve glucose control over time [15].

With the aim of investigating glucose patterns associated with COVID-19, we studied CGM in patients hospitalized for COVID-19 and mild-to-moderate pneumonia, regardless of a possible accompanying diagnosis of diabetes, and searched for possible associations with clinical features and outcome of the disease.

## Subjects, materials and methods

### Participants

Prospective observational study that took place at San Raffaele Hospital in Milan (Italy), a referral center for Covid-19 patients in the Lombardy Region. We enrolled consecutive adult patients admitted at the hospital for SARS-CoV-2 infection, from April 13th, 2020 to May 18^ h^, 2020 and from November 1st, 2020 to January 6th, 2021, during the first two pandemic waves occurred in Italy. Criteria of inclusion were diagnosis of Covid-19 pneumonia, admission to Internal Medicine Department, age > 18 years, ability to give informed consent. Diagnosis of Covid-19 associated pneumonia was based on reverse-transcriptase polymerase chain reaction (RT-PCR) positivity for SARS-CoV-2 on nasopharyngeal swab and typical radiological findings at chest X-ray. Both patients with and without diabetes were included. Pregnant women were excluded.

Full anthropometric, clinical and laboratory data were obtained from clinical records. Pertinent to this study were the following laboratory and clinical parameters: fasting glucose, glycated haemoglobin (HbA1c), insulin and C-peptide; C-reactive protein (CRP), blood cells count, interleukin 6 (IL-6), and D-dimer. Insulin resistance HOMA index was also calculated [16].

With regard to Covid infection and associated pneumonia patients were treated according to hospital protocol for standard of care applied during hospitalization, as reported [5]. Standard clinical outcomes measured during the study included duration of hospitalization before discharge, need of intubation and/or transfer to intensive care unit (ICU) or death.

#### CGM

All patients, regardless of their possible concomitant diagnosis of diabetes, inserted a blinded glucose sensor (Envision™ Pro, Medtronic Minimed, Northridge, CA 91325, USA) that recorded interstitial glucose levels. Sensors were applied at the admission to the hospital ward and worn for 7 days. They were fully disposable, required no calibration and lasted up to one week. Glucose sensor devices were applied by trained health care providers at patient bedside. Glucose values were automatically uploaded on Carelink™ (Medtronic) web software with a professional hospital account and data were recorded in a dedicated electronic case report form. Patients were blind to glucose measurements by the sensor. The following glucose sensor metrics were calculated over the entire period of sensor wearing: mean and median glucose, standard deviation (SD), coefficient of variation (CV), time in glucose range (TIR 70–180 mg/dl) and time in tight glucose range (TITR 70–140 and), time below range (TBR < 70 mg/dl), time above range (TAR > 140, > 180) [17, 18].

### Respiratory function measures

Respiratory function parameters were recorded daily as arterial partial pressure of oxygen (PaO_2_) to fraction of inspired oxygen (FiO_2_) ratio (PaO_2_:FiO_2_) and need of non-invasive ventilation (NIV).

Respiratory function was also graded into four categories based on PaO_2_:FiO_2_ according to the Berlin definition of acute respiratory distress syndrome (ARDS): severe respiratory failure (≤ 100 mmHg), moderate respiratory failure (> 100 and ≤ 200 mmHg), mild hypoxemia (> 200 and ≤ 300 mmHg), normal respiratory function (> 300 mmHg) [19].

### Chest X-rays

Chest X-rays were performed on admission and after the removal of glucose sensors (1 week) and were analyzed both by expert radiologists and artificial intelligence in order to score pneumonia severity. Radiographic Assessment of Lung Edema (RALE) score [20] was used to quantify areas of lung opacities in each radiographic quadrant. Artificial intelligence (AI) software (qXR v2.1, Qure.ai Technologies, India) was used to measure X-rays density expressed as QURE AI scores.

### Study outcomes

Outcome of the study was the relation of glucose, measured according to the different CGM metrics, with respiratory function (primary), clinical outcomes and radiological scores of chest X-rays (secondary). Respiratory function was defined as improving, stable or worsening in case of change, or lack of change, in the PaO_2_:FiO_2_ category scale of the ARDS definition reported above.

### Ethics

The study is a part of COVID-BioB Study, approved by the local Ethics Committee (NCT04318366), and obtained specific approval for glucose sensor use. Patients signed informed consent for glucose sensor insertion. Research was performed in accordance with the Helsinki Declaration.

### Statistical analysis

Categorical variables are presented as numbers and percentages, continuous variables as median and interquartile range (IQR) according to the type of distribution. Continuous variables were compared between groups using Wilcoxon’s test. Categorical variables were compared between groups using Chi-square test or Fisher’s exact test, as appropriate. The possible predictors of the glycemic outcomes were studied using the correlation statistics. The Pearson correlation test was performed to assess the statistical significance of each correlation. Multivariate regression models were used to further study possible relationship between respiratory condition and glycaemic outcomes. All statistical tests were based on a two-sided significance level of 0.05. Statistical analysis was performed using R software, version 4.2.1, (R Foundation for Statistical Computing, Vienna, Austria).

## Results

### Baseline patient characteristics and impact of steroid treatment

Forty-six patients were enrolled in the study, 22 patients during the first pandemic wave and 24 during the second one. We excluded from final analysis 3 patients (1 from the first wave and 2 from the second wave) due to the absence of data recorded by CGM. The majority of patients were male (n = 29, 67.4%) and Caucasian (n = 35, 81.4%). Eight patients (18.6%) had a known diagnosis of T2D, none had T1D and none used glucose sensors before study admission. Median age was 65 years (inter-quartile range [IQR] 58–60). A large proportion of patients were overweight (n = 21, 48.8%). General characteristics of patients at admission, their biochemical, radiological and clinical outcomes and glucose measures are shown in Table 1. Time of CGM wearing was in median 7 [IQR 7–8] days. The sub-group of patient with known diabetes (18.6%) was similar to patients without diabetes as general characteristics, BMI, comorbidities and respiratory function (Table 1). Participants from the 1st and 2nd wave subgroups were compared, showing some minor differences: higher weight, BMI and rate of steroid treatment already ongoing at time of hospitalization in all 2nd wave patients at time of hospitalization; higher C-peptide and leukocyte count in the 2nd wave subgroup, possibly reflecting steroid treatment effect; a marginal worse QURE AI score (higher X-rays density) in 1st wave subgroup; a worse respiratory function (lower PaO_2_:FiO_2_) in the 2nd wave group. No differences were observed between the two subgroups with regard to need for NIV, ICU or intubation, death and duration of hospitalization. Patients treated with steroids showed higher average glucose levels (p = 0.0083) and CV (p = 0.05) and lower TITR 70–140 (p = 0.0074) (Supplementary information 1 and 2).

**Table 1:** General characteristics, clinical and radiological outcomes and glucose measures of patients at admission at the hospital

Number	43
Age (years)	65 (58–70)
Male	29 (67.4)
Ethnicity	
Caucasian	35 (81.4)
Latino	5 (11.6)
Asian	1 (2.3)
Austronesian	2 (4.7)
Weight (kg)	78 (70–90)
Height (cm)	170 (163–177)
BMI (Kg/m^2^)	
BMI < 18.49	0
18.5 < BMI < 24.99	12 (27.91)
25 < BMI < 29.99	21 (48.8)
BMI > 30	10 (23.3)
Known type 2 diabetes	8 (18.6)
Hypertension	25 (58.1)
Coronary artery disease	7 (16.3)
Chronic Renal failure	3 (7.0)
Chronic obstructive pulmonary disease	6 (14.0)
Cancer	5 (11.6)
Insulin therapy during hospitalization in known diabetes	7 (87.50)
Insulin therapy during hospitalization in not previously known diabetes	4 (11.43)
Corticosteroids	28 (65.1)
COVID-19 specific therapy:	
None	22 (51.2)
Hydroxychloroquine	11 (25.6)
Remdesivir	5 (11.6)
AMY101 (complement inhibitor)	1 (2.3)
Hydroxychloroquine + remdesivir + Ritonavir/Lopinavir	1 (2.3)
Hydroxychloroquine + Ritonavir/Lopinavir	2 (4.7)
Hydroxychloroquine + remdesivir	1 (2.3)
Anakinra	9 (20.9)
HbA1c at admission (mmol/mol)	42.5 (39–48)
Ranges of HbA1c at admission:	
< 42 mmol/mol	17 (39.5)
42–48 mmol/mol	14 (32.6)
> 48 mmol/mol	11 (25.6)
Glucose at admission (mg/dl)	96 (83–134)
Insulinemia at admission (mU/L)	12.7 (7.3–16.5)
HOMA index	3.1 (1.76–4.36)
Fasting C peptide at admission (ng/mL)	3.18 (2.13–4.27)
CRP at admission (mg/L)	60 (25–130)
WBC at admission (10^9/L)	7.3 (5.9–11.5)
Linfocyte at admission (10^9/L)	0.9 (0.7–1.5)
IL6 at admission (pg/mL)	38.7 (14.5–57.75)
D-dimer at admission (mcg/mL)	0.71 (0.42–1.44)
Need for NIV	20 (46.5)
Need for ICU	8 (18.6)
Need for intubation	5 (11.6)
Death	6 (14.0)
Days of hospitalization	14 (10–22)
QURE at admission	39 (18.5–57.5)
QURE after CGM removal	52.5 (36–73)
RALE at admission	6 (2–12)
RALE after CGM removal	14 (7–18)
PaO_2_:FiO_2_ at admission	249 (112–306)
PaO_2_:FiO_2_ after CGM removal	257 (118–363)
Respiratory function at baseline:	
Normal	12 (27.9)
Mild Hypoxemia	13 (30.2)
Moderate Respiratory failure	10 (23.3)
Severe respiratory failure	8 (18.6)
Respiratory function after CGM removal:	
Normal	17 (39.5)
Mild Hypoxemia	8 (18.6)
Moderate Respiratory failure	11 (25.6)
Severe respiratory failure	7 (16.3)
Change in respiratory function:	
Worsening	10 (23.3)
Stable	17 (39.5)
Improvement	16 (37.2)
Average CGM glucose mg/dL	120 (110–153)
SD	21 (16–34)
CV	18.7 (13.7–21. 2)
Time below range < 70 mg/dL (%)	0 (0–0)
Time in range 70–180 mg/dL (%)	98 (79.5–99)
Time above range > 180 mg/dL (%)	2 (0.5–20.5)
Time in tight range 70–140 mg/dL (%)	84 (41.5–94)
Time above range > 140 mg/dL (%)	16 (6–58.5)

Continuous variables reported as median and interquartile range (IQR), categorical variables reported as number and percentages

*BMI* body mass index, *T2 diabetes* type 2 diabetes, *SGLT2 i* sodium-glucose co-transporter 2 (sglt2) inhibitors, *DDPIV i* dipeptidyl peptidase-IV inhibitors, *CGM* continuous glucose monitoring, *CRP* C reactive protein, *WBC* white blood cells, *IL6* interleukin 6, *RALE* Radiographic Assessment of Lung Edema, *PaO*_*2*_*:FiO*_*2*_ arterial partial pressure of oxygen (PaO_2_) to fraction of inspired oxygen (FiO_2_) ratio, *NIV* non-invasive ventilation, ICU intensive care unit, *SD* standard deviation, *CV* coefficient of variation

### CGM and respiratory function

**Fig. 1:**
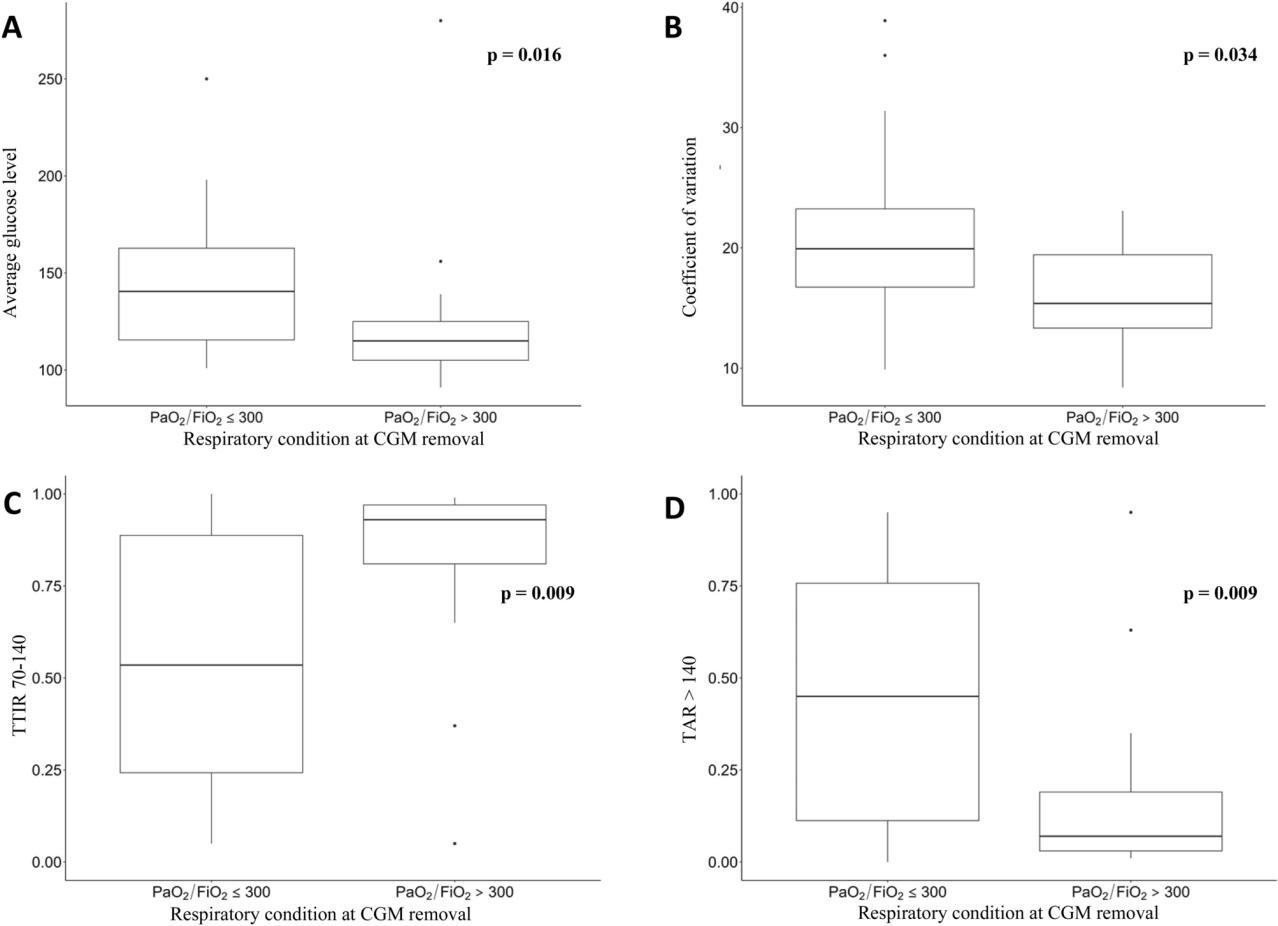
Box plots for glycemic metrics by respiratory condition at time of CGM removal. **A** average glucose levels. **B** Coefficient of variation of glucose. **C** Time in tight range 70–140 mg/dL. **D** Time above range > 140 mg/dL

During the 7 days of CGM recording, respiratory function, expressed as PaO_2_:FiO_2_ ratio and classified according to the four category scale, worsened in 10 patients, remained stable in 17 and improved in 16. At the time of sensor removal PaO_2_:FiO_2_ ratio resulted positively correlated with TIR 70–180 (r = 0.32, p < 0.05) and TITR 70–140 (r = 0.49, p < 0.001), while it was negatively correlated with average glucose (r =– 0.31, p < 0.05), CV (r =– 0.47, p < 0.01) and TAR > 140 (r =– 0.49, p < 0.001). No relations were observed between respiratory function outcome and HbA1c. Patients with normal respiratory function (PaO_2_:FiO_2_ ratio > 300) at time of sensor removal, showed significantly lower average glucose, CV and TAR > 140 and higher TITR 70–140 (Fig. 1).

The relationship between respiratory function and glucose metrics, evaluated by the multivariate regression model showed that, regardless of the respiratory condition at baseline, a normal respiratory function at time of CGM removal positively correlated with TITR 70–140 mg/dL (p < 0.01) and negatively correlated with CV and TAR > 140 mg/dL (both p < 0.05). No significant correlation was observed with average glucose and TIR 70–180 mg/dL (Table 2).

**Table 2:** Multiple regression model for normal respiratory function

	Average glucose level (SD)	Coefficient of variation glucose level (SD)	TITR 70–140 (SD)	TIR 70–180 (SD)	TAR > 140 (SD)
PaO_2_/FiO_2_ > 300 at baseline	– 2.38 (13.94)	– 0.91 (2.17)	– 3.56 (10.79)	– 0.07 (7.86)	3.36 (10.79)
PaO_2_/FiO_2_ > 300 at time of CGM removal	– 19.59 (12.79)	– 4.28* (1.99)	26.93** (9.90)	12.67 (7.21)	– 26.75* (9.90)

*SD* standard deviation, *TIR* time in range, *TITR* time in tight range, *TAR* time above range, *CGM* continuous glucose monitoring

^***^ p < 0.001; ** p < 0.01; * p < 0.05

### CGM and chest X-ray

CV was positively correlated with RALE score at time of CGM removal (r = 0.35, p < 0.05), while no other correlations were observed between glucose metrics, HbA1c, RALE and QURE AI scores.

### CGM and clinical outcomes

Several CGM values had a significant correlation with duration of hospitalization: in detail, average glucose, CV and TAR > 140 had a positive correlation with duration of hospitalization (r = 0.35, p < 0.05; r = 0.49, p < 0.001, r = 0.48, p < 0.01 respectively), while TITR 70–140 and TIR 70–180 had a negative correlation (r =– 0.48, p < 0.01, r =– 0.41, p < 0.01, respectively). No other significant correlations between CGM metrics and clinical outcome, including need of intubation and/or transfer to ICU or death, were observed, possibly due to small numbers.

## Discussion

Hyperglycaemia has long been identified, both in people with or without diabetes, as a risk factor for a worse prognosis in patients with COVID-19 disease, with a risk proportionally increased with elevated glucose and HbA1c in people with diabetes [12–14, 21–27]. The pathophysiological mechanisms responsible for the detrimental role of glucose, within and outside diabetes, still deserve investigations. We used CGM for one week in a cohort of 43 consecutive patients hospitalized for COVID-19 disease with mild-to-moderate pneumonia, regardless of glucose levels and a possible accompanying diagnosis of diabetes at hospital admission. The aim was to evaluate the impact of different glucose patterns and ranges measured by CGM metrics on respiratory function and, more in general, clinical outcome.

Our findings confirmed glucose as a negative prognostic marker of clinical outcome: CGM-derived higher average glucose and glucose variability were associated with lower values of PaO_2_:FiO_2_ ratio and longer duration of hospitalization of COVID-19 patients, while more time spent in tighter glucose control, as reflected by higher TITR 70–140, resulted in better respiratory function and shorter hospitalization. Regardless of respiratory function at admission, patients with normal PaO_2_:FiO_2_ ratio at the time of CGM removal showed lower glucose variability, measured as CV, and higher time spent in TITR 70–140. Average glucose and TIR 70–180 showed no association with respiratory function at the moment of CGM removal, confirming that lower values of glucose are prognostically beneficial.

The observation that increased TAR > 140 is associated with a worse clinical and respiratory prognosis reinforce the evidence of the unhealthy role of glucose abnormalities, even of modest entity. With regard to cardiovascular diseases, the increased risk associated with minor elevations of glucose is well established [28]. With regard to infections, it was shown that poor glycemic control is a strong risk factor in patients with diabetes [29], but few data are available about a possible impact of minor glucose abnormalities on risk and prognosis of infectious diseases. Our findings, from a cohort largely represented by non-diabetic patients, show a detrimental role of even slightly elevated blood glucose on the outcome of mild-to-moderate pneumonia associated with COVID-19 infection. Although this observation from a small patient sample cannot be generalized, is intriguing.

Interestingly, no association was observed with HbA1c, possibly indicating that, whatever is the pathophysiological role played by glucose, this is reflected by its current, rather than preceding trend. HbA1c mirrors glycaemic values of the last 2–3 months before infection, while CGM-derived glycaemic metrics reflect current glucose control during hospitalization; this may point out that in-hospital glucose control is more important for a favourable prognosis than glucose control prior to hospital admission. This result is consistent with previous reports [30, 31] showing that preceding long-term glucose control is not correlated with clinical outcomes of diabetic patients hospitalized for COVID-19 infection.

The only association of a glycaemic metric with radiological severity scores was between CV and RALE, while no correlations were shown for the remaining glucose metrics and RALE and any of them with QURE AI scores. This finding suggests the primacy, or at least a relevant role, of glucose variability in the hierarchy of glucose abnormalities as associated risk factors to the pathophysiology of COVID-19 pneumonia [32], and emphasizes its possible detrimental role in infections similar to that claimed in cardiovascular complications [33].

Although the topic of glucose control in relation to Covid-19 outcomes has been described by previous studies, few others used glucose monitoring for investigating the correlation of glycaemic metrics and COVID-19 infection severity [34, 35]. In a Chinese series of patients with diabetes and COVID-19, an increased risk of adverse outcomes was shown with glucose levels > 160 mg/dL, < 70 mg/dL and high CV [35]. In our study, in a cohort of persons with and without diabetes, TITR emerged as a strong predictor of better prognosis, measured either as respiratory function or clinical outcome. TITR is considered as a new parameter to evaluate dysglycemia in diabetes [36], new reasonable treatment target for young patients [37] and possible threshold to identify progressors to clinical type 1 diabetes in at risk individuals [38]. A recent international Consensus suggested TITR as an outcome in research studies using CGM-derived data [39].

The main limitation of our study is the small size of the population investigated, nonetheless comparable with the few other studies investigating CGM in COVID-19 patients. The small number of patients and of serious adverse events (admission to ICU and death) does not allow to perform analysis on these hard outcomes. Moreover, the disparity of steroid use between 1st and 2nd wave groups was responsible for differences in blood glucose, higher in steroid treated patients: however, this was a minor imbalance and did not affect the correlation between glucose metrics and clinical outcomes, both considering separated or together the two groups. Study took place in 2020–2021, when the Covid-19 variant was different from the current circulating variants. But our cohort was limited to mild-to-moderate pneumonia cases that can reflect most of the SARS-CoV-2 recent variants’ infections that are now mild in severity, with low overall mortality.

The relation of glucose abnormalities and severity of COVID infection has been extensively investigated since the beginning of pandemic. Our findings add the evidence that lower glucose variability, measured as CV, and optimal glucose control, expressed as higher TITR 70–140 mg/dL, are CGM measurable metrics predictive of a better prognosis in COVID-19 patients with mild-to-moderate pneumonia.

## Supplementary Information

Below is the link to the electronic supplementary material.

Supplementary file1 (PPTX 108 KB)

Supplementary file2 (DOCX 19 KB)
