# Supplementary material for: Tight and stable glucose control is associated with better prognosis in patients hospitalized for Covid-19 and pneumonia
Source: Acta Diabetol. 2024 Nov 29;62(6):925–33. doi: 10.1007/s00592-024-02409-8 (PMC12141156; doi:10.1007/s00592-024-02409-8)
Supplement: Supplementary file 1 — Supplementary file1 (PPTX 108 KB) [file 592_2024_2409_MOESM1_ESM.pptx]

## Slide 1
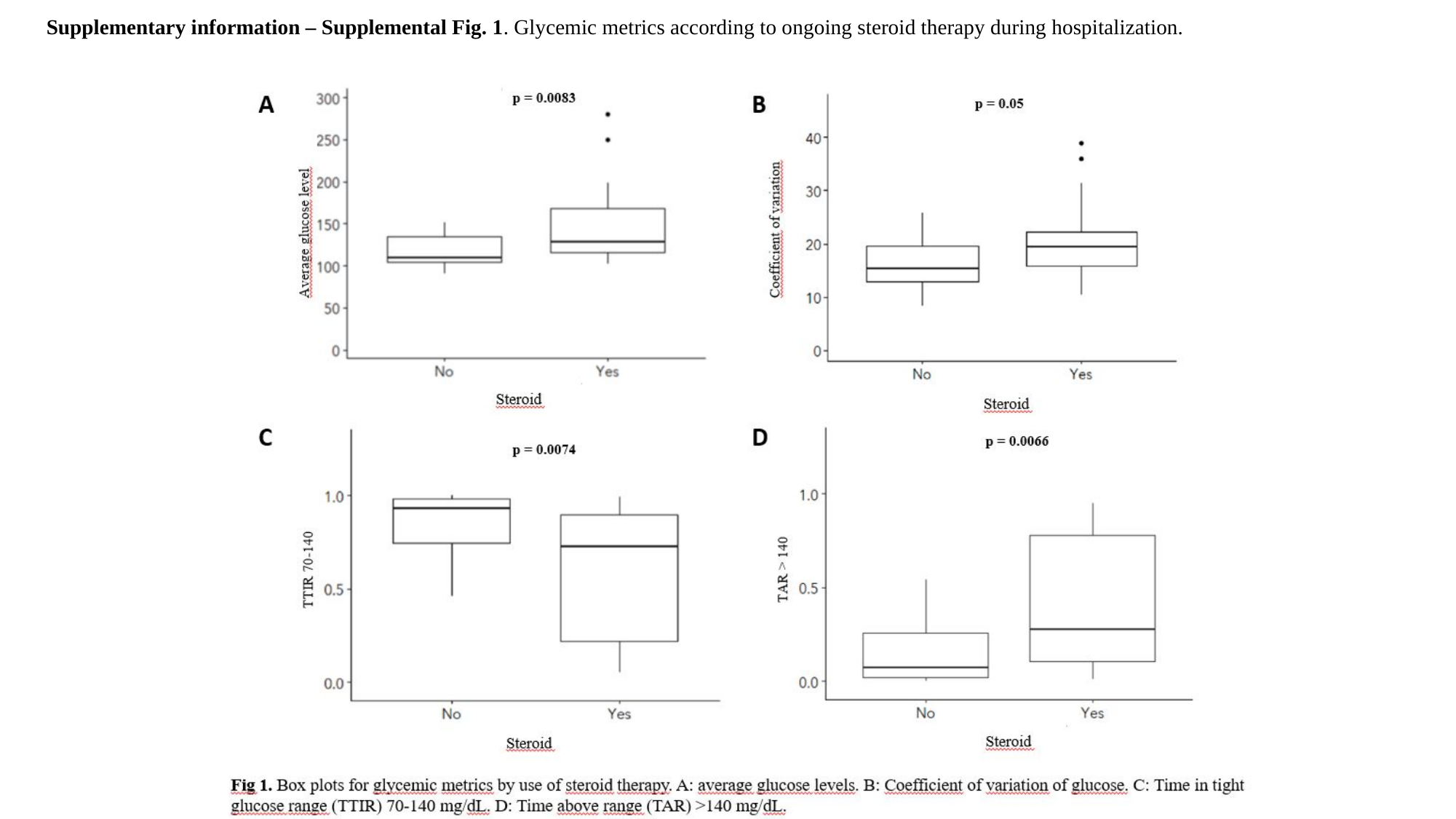

Supplementary information – Supplemental Fig. 1. Glycemic metrics according to ongoing steroid therapy during hospitalization.
