# Supplementary material for: Tight and stable glucose control is associated with better prognosis in patients hospitalized for Covid-19 and pneumonia
Source: Acta Diabetol. 2024 Nov 29;62(6):925–33. doi: 10.1007/s00592-024-02409-8 (PMC12141156; doi:10.1007/s00592-024-02409-8)
Supplement: Supplementary file 2 — Supplementary file2 (DOCX 19 KB) [file 592_2024_2409_MOESM2_ESM.docx]

**Supplementary material 2 - Table 1**. Baseline general characteristics, clinical and radiological outcomes and glucose measures of patients at admission at the hospital, in participants from 1^st^ vs 2^nd^ pandemic waves.

|  | **1st wave** | **2nd wave** | **p** |
| --- | --- | --- | --- |
| Number | 21 | 22 |  |
| Age (years) | 66 (53-70) | 64.5 (63-69) | 0.478 |
| Male | 12 (57.1) | 17 (77.3) | 0.203 |
| Etnicity |  |  | 0.129 |
| caucasian | 16 (76.2) | 19 (86.4) |  |
| latino | 4 (19.0) | 1 (4.5) |  |
| asian | 1 (4.8) | 0 |  |
| austronesian | 0 | 2 (9.1) |  |
| Weight (kg) | 70 (64-78) | 86 (77-94) | **0.006** |
| Height (cm) | 170 (160-175) | 170 (165-178) | 0.293 |
| BMI (Kg/m^2^) |  |  |  |
| BMI <18.49 | 0 | 0 | **0.002** |
| 18.5< BMI <24.99 | 11 (52.4) | 1 (4.5) |  |
| 25< BMI <29.99 | 7 (33.3) | 14 (63.6) |  |
| BMI >30 | 3 (14.3) | 7 (31.8) |  |
| Known type 2 diabetes | 5 (23.8) | 3 (13.6) | 0.457 |
| Hypertension | 11 (52.4) | 14 (63.6) | 0.543 |
| Coronary artery disease | 4 (19.0) | 3 (13.6) | 0.698 |
| Chronic Renal failure | 3 (14.3) | 0 | 0.108 |
| Chronic obstructive pulmonary disease | 5 (23.8) | 1 (4.5) | 0.095 |
| Cancer | 3 (14.3) | 2 (9.1) | 0.664 |
| Insulin therapy during hospitalization in known diabetes | 4 (80.00) | 3 (100) | 1.000 |
| Insulin therapy during hospitalization in not previously known diabetes | 1 (6.25) | 3 (15.79) | 0.608 |
| Corticosteroids | 6 (28.6) | 22 (100) | **<0.001** |
| COVID-19 specific therapy: |  |  | **<0.001** |
| None | 4 (19.0) | 18 (81.8) |  |
| hydroxychloroquine | 11 (52.4) | 0 |  |
| remdesivir | 1 (4.8) | 4 (18.2) |  |
| AMY101 (complement inhibitor) | 1 (4.8) | 0 |  |
| hydroxychloroquine + remdesivir + Ritonavir/Lopinavir | 1 (4.8) | 0 |  |
| hydroxychloroquine + Ritonavir/Lopinavir | 2 (9.5) | 0 |  |
| hydroxychloroquine + remdesivir | 1 (4.8) | 0 |  |
| Anakinra | 2 (9.5) | 7 (31.8) | 0.097 |
| HbA1c at admission (mmol/mol) | 42 (38-48) | 43 (40-45) | 0.269 |
| Ranges of HbA1c at admission:  <42 mmol/mol  42-48 mmol/mol  >48 mmol/mol | 9 (42.9)  6 (28.6)  6 (28.6) | 8 (36.4)  8 (36.4)  5 (22.7) | 0.857 |
| glucose at admission (mg/dl) | 86 (82-101) | 106 (95-136) | 0.241 |
| insulinemia at admission (mU/L) | 10.3 (5.9-15.3) | 13.6 (11.1-18.2) | 0.088 |
| HOMA index | 2.23 (1.5-3.47) | 3.77 (2.8-7.34) | 0.057 |
| fasting C peptide at admission (ng/mL) | 2.31 (1.86-3.19) | 3.78 (3.18-4.46) | **0.018** |
| CRP at admission (mg/L) | 43 (14-123) | 98 (37-131) | 0.239 |
| WBC at admission (10^9/L) | 7 (5.5-8.6) | 9.7 (6.4-13.5) | **0.045** |
| linfocyte at admission (10^9/L) | 1 (0.9-1.5) | 0.85 (0.5-1.6) | 0.489 |
| IL6 at admission (pg/mL) | 38.9 (21.2-55.8) | 22.6 (14.5-60.6) | 0.341 |
| D-dimer at admission (mcg/mL) | 0.64 (0.42-1.54) | 0.78 (0.44-1.36) | 0.339 |
| need for NIV | 7 (33.3) | 13 (59.1) | 0.129 |
| need for ICU | 2 (9.5) | 6 (27.3) | 0.240 |
| need for intubation | 1 (4.8) | 4 (18.2) | 0.345 |
| death | 2 (9.5) | 4 (18.2) | 0.664 |
| days of hospitalization | 13 (8-19) | 16 (11-26) | 0.298 |
| QURE at admission | 56 (0-92) | 32 (20.5-48) | 0.058 |
| QURE after CGM removal | 78 (30-111) | 49.5 (40-60.8) | **0.044** |
| RALE at admission | 6.5 (0.5-12.5) | 6 (3-9) | 0.770 |
| RALE after CGM removal | 11.5 (5-18) | 15 (8-17.5) | 0.875 |
| PaO2:FiO2 at admission | 279 (250-318) | 132 (84-247) | **<0.001** |
| PaO2:FiO2 after CGM removal | 318 (195-381) | 187 (94-321) | **0.022** |
| Respiratory function at baseline:  Normal  Mild Hypoxemia  Moderate Respiratory failure  Severe respiratory failure | 10 (47.6)  8 (38.1)  2 (9.5)  1 (4.8) | 2 (9.1)  5 (22.7)  8 (36.4)  7 (31.8) | **0.003** |
| Respiratory function after CGM removal:  Normal  Mild Hypoxemia  Moderate Respiratory failure  Severe respiratory failure | 11 (52.4)  4 (19)  6 (28.6)  0 (0) | 6 (27.3)  4 (18.2)  5 (22.7)  7 (31.8) | **0.031** |
| Change in respiratory function:  Worsening  Stable  Improvement | 6 (28.6)  9 (42.9)  6 (28.6) | 4 (18.2)  8 (36.4)  10 (45.5) | 0.537 |
| average CGM glucose mg/dL | 115 (105-140) | 129 (117-159) | 0.059 |
| SD | 18 (14-28) | 25.5 (20-38) | **0.040** |
| CV | 16.51 (13.3-20.2) | 19.4 (16.0-38.9) | 0.052 |
| Time below range <70 mg/dL (%) | 0 (0-0) | 0 (0-0) | 0.834 |
| Time in range 70-180 mg/dL (%) | 99 (88-100) | 95 (71-99) | 0.242 |
| Time above range >180 mg/dL (%) | 1 (0-12) | 5 (1-29) | 0.164 |
| Time in tight range 70-140 mg/dL (%) | 93 (59-98) | 72.5 (23-88) | 0.080 |
| Time above range >140 mg/dL (%) | 7 (2-41) | 27.5 (12-77) | 0.078 |

Continuous variables reported as median and interquartile range (IQR), categorical variables reported as number and percentages.

BMI= body mass index. T2 diabetes= type 2 diabetes. SGLT2 i= sodium-glucose co-transporter 2 (sglt2) inhibitors. DDPIV i= dipeptidyl peptidase-IV inhibitors. CGM= continuous glucose monitoring. CRP= C reactive protein. WBC= white blood cells. IL6= interleukin 6. RALE= Radiographic Assessment of Lung Edema. PaO2:FiO2= arterial partial pressure of oxygen (PaO2) to fraction of inspired oxygen (FiO2) ratio. NIV= non-invasive ventilation. ICU= intensive care unit. SD= standard deviation. CV= coefficient of variation.
